# Supplementary material for: Different Types of Hypericum perforatum cvs. (Elixir, Helos, Topas) In Vitro Cultures: A Rich Source of Bioactive Metabolites and Biological Activities of Biomass Extracts
Source: Molecules. 2023 Mar 4;28(5):2376. doi: 10.3390/molecules28052376 (PMC10005045; doi:10.3390/molecules28052376)

Figure S1. Free radical scavenging activity (DPPH test) of the methanol extracts obtained from biomass of *in vitro* agitated cultures of the *H. perforatum* cultivars Helos, Elixir, and Topas (MS, 0.1 mg/L BAP and 0.1 mg/L NAA, 5 weeks). Values are expressed as the mean ± SD (n = 3).

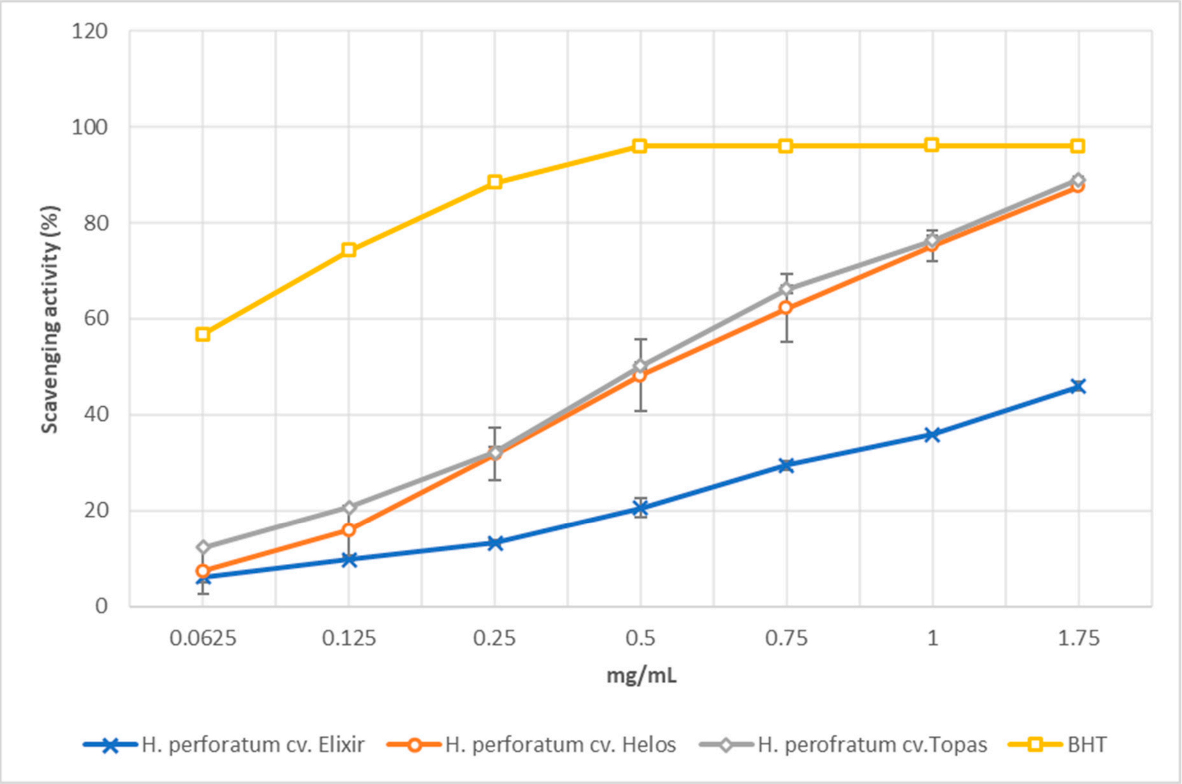

Figure S2. Reducing power of the methanol extracts obtained from biomass of *in vitro* agitated cultures of the *H. perforatum* cultivars Helos, Elixir, and Topas (MS, 0.1 mg/L BAP and 0.1 mg/L NAA, 5 weeks), evaluated by spectrophotometric detection based on the Fe^{3+}-Fe^{2+} transformation method. Values are expressed as the mean ± SD (n = 3).

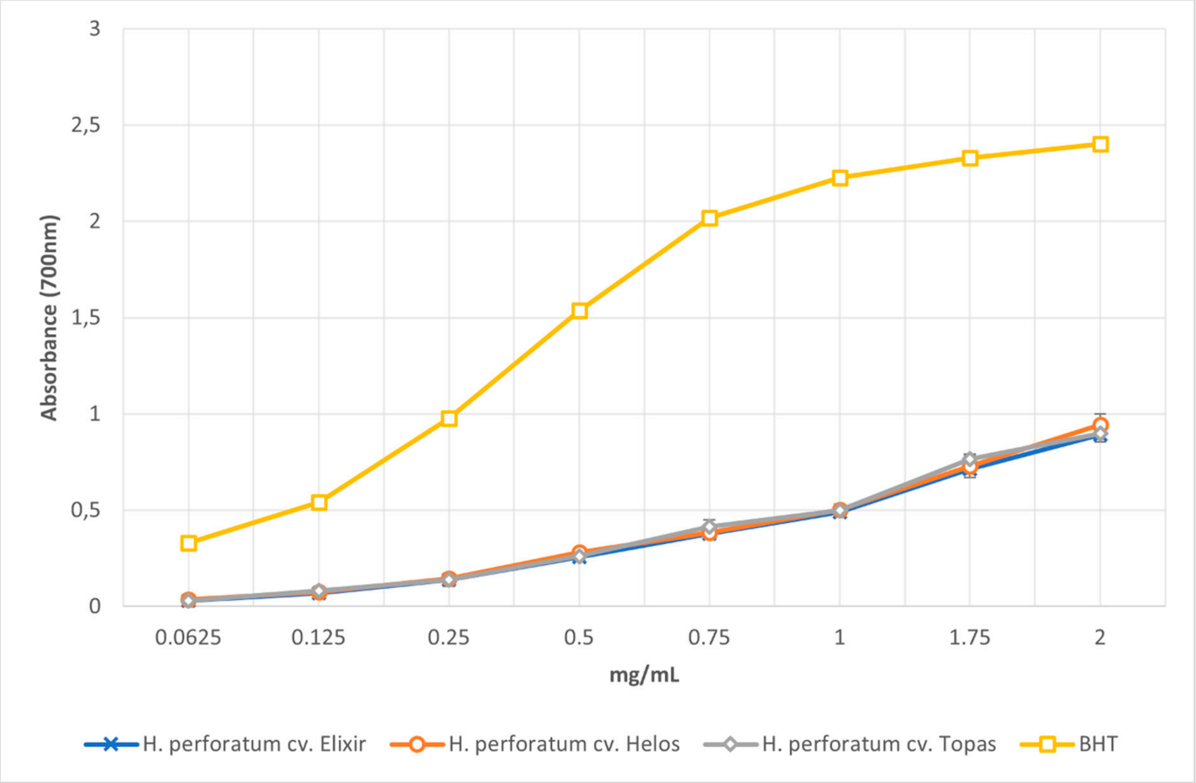

Figure S3. The chelating activity of the methanol extracts obtained from biomass of *in vitro* agitated cultures of the *H. perforatum* cultivars Helos, Elixir, and Topas (MS, 0.1 mg/L BAP and 0.1 mg/L NAA, 5 weeks), measured by inhibition of ferrozine-Fe^{2+} complex formation. Values are expressed as the mean ± SD (n = 3).

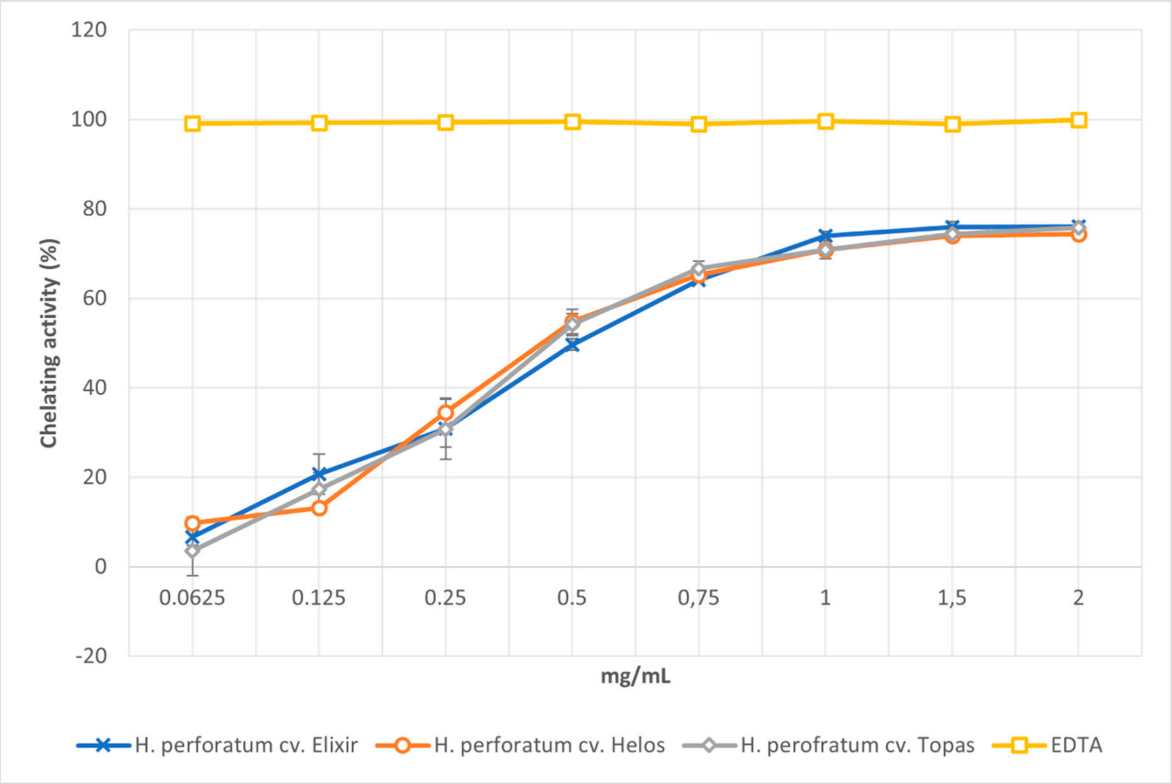

Supplement: Supplementary file 1 [file molecules-28-02376-s001.zip › molecules-2153913-supplementary.pdf]
